# Neuropsychiatric, stress-related symptoms and essential tremor clinical variability

**DOI:** 10.1007/s00702-025-03026-7

**Published:** 2025-10-06

**Authors:** Giulia Paparella, Matteo Panfili, Sena Akgun, Luca Angelini, Adriana Martini, Anna Sofia Grandolfo, Martina De Riggi, Davide Costa, Daniele Birreci, Annalisa Maraone, Francesco Saverio Bersani, Matteo Bologna

**Affiliations:** 1 IRCCS Neuromed, Pozzilli, IS Italy; 2 Neurophysiopathology Unit, Department of Translational Biomedicine and Neuroscience, University of Bari Aldo Moro, Bari, Italy; 3 Department of Human Neurosciences, Sapienza University of Rome, Rome, Italy; 4 Department of Medico-Surgical Sciences and Biotechnologies, Sapienza University of Rome, Rome, Italy

**Keywords:** Essential tremor, Psychiatric symptoms, Stress, Stress-related disorders, Sleep disorders

## Abstract

Essential tremor (ET) is characterized by marked clinical variability, possibly influenced by factors such as neuropsychiatric comorbidities that elevate stress levels. Psychological stress frequently exacerbates tremor severity, establishing a feedback loop that intensifies functional impairments and social consequences, ultimately increasing the overall disease burden. Here we aim to examine the relationship between neuropsychiatric disorders and stress-related symptoms on tremor variability in ET patients. Forty-seven ET patients underwent neurological and psychiatric assessments, including standardized scales. Data were analyzed with non-parametric tests, and Spearman’s correlation was used to explore relationships between demographics and clinical measures. Concomitant psychiatric disorders (also including mood and personality disorders) were identified in 23 out of 47 patients (48.9%). Patients showed elevated levels of perceived stress. Insomnia, reported by 20 patients (42.6%), was significantly associated with increased tremor severity, as reflected in total tremor scores and measures related to activities of daily living (P values < 0.001). No other significant correlations were observed between clinical or demographic variables. In ET, we observed a high frequency of neuropsychiatric disorders and elevated stress levels. Tremor severity was associated with insomnia although it did not correlate with stress or psychiatric symptoms. This finding may reflect shared mechanisms between tremor and sleep disturbances in ET, possibly involving the locus coeruleus.

## Introduction

Essential tremor (ET) is one of the most prevalent movement disorders, affecting millions of individuals worldwide (Louis 2011; Bhatia et al. 2018). ET is characterized by an action (postural or kinetic) tremor of the upper limbs, possibly associated with tremor in other body parts, including lower limbs, head and voice (Bhatia et al. 2018). ET has increasingly been recognized as a condition with considerable clinical heterogeneity (Louis 2011, 2020; Hopfner and Deuschl 2018; Bologna et al. 2019; Erro et al. 2022). This variability regards the motor manifestations of the disease, e.g., tremor distribution, severity, possible progression, and the association with other subtle neurological motor signs besides tremor (Bologna et al. 2019, 2020; Louis 2020; Pandey and Bhattad 2021; McGurn et al. 2022; Angelini et al. 2022; Erro et al. 2024; Paparella et al. 2024a, b).

Among the non-motor symptoms of ET, psychiatric conditions—particularly anxiety and depression (Benito-León et al. 2006; Lorenz et al. 2011; Lee et al. 2015; Lenka et al. 2017; Smeltere et al. 2017; Bologna et al. 2019; Dai et al. 2022; Gerbasi et al. 2022; Ratajska et al. 2022; Angelini et al. 2022; Berry et al. 2024; Paparella et al. 2024b), and to a less extent personality disturbances (Chatterjee et al. 2004; Lorenz et al. 2006; Bologna et al. 2019; Angelini et al. 2022), are more prevalent than in the general population, significantly contributing to both symptom variability and the overall disease burden (Lombardi et al. 2001; Benito-León et al. 2006; Lorenz et al. 2011; Lee et al. 2015; Lenka et al. 2017; Smeltere et al. 2017; Dai et al. 2022; Gerbasi et al. 2022; Ratajska et al. 2022; Angelini et al. 2022; Berry et al. 2024; Paparella et al. 2024b). Multiple studies have shown that anxiety or depression in ET are not simply psychological reactions to the presence or disabling effects of tremor (Lenka et al. 2017; Bologna et al. 2019). Accordingly, their severity does not correlate with tremor severity (Lenka et al. 2017; Bologna et al. 2019; Angelini et al. 2022). In fact, cross-sectional and prospective data demonstrated that baseline self-reported depression, as well as the use of antidepressants, are associated with an increased risk of developing ET (Louis et al. 2007). Furthermore, anxiety, depression, and other psychiatric symptoms such as social phobia, are linked in ET to greater functional disability, and this relation, again, is independent of tremor severity (Schneier et al. 2001; Louis et al. 2001; Ozel-Kizil et al. 2008). Finally, mood symptom severity appears to be associated with cognitive impairment in ET patients (Ratajska et al. 2022).

The above mentioned psychiatric conditions are characterized by increased levels of perceived psychological stress, which in turn may exacerbate tremor and other symptoms (Koller et al. 1989; Schneier et al. 2001; Louis et al. 2001; Tan et al. 2005; Handforth and Parker 2018; Russell and Lightman 2019; Dai et al. 2022; O’Suilleabhain et al. 2023). The mechanisms underlying the relationship between stress and tremor exacerbation, specifically, why stress increases tremor or whether tremor patients are more sensitive to stress, are not yet fully understood. These mechanisms may involve stress hormones such as adrenaline, noradrenaline, and cortisol, which increase muscle tension and heart rate, potentially triggering or worsening tremor, and also exert effects on the central nervous system (Dirkx et al. 2020; van der Heide et al. 2021). Patients with ET are highly sensitive to psychological stress, which has been linked to both the onset and progression of their symptoms. Accordingly, propranolol—a first-line, non-selective beta-adrenergic blocker—exerts its therapeutic effects by attenuating not only tremor but also associated symptoms of anxiety, agitation, and stress (Paparella et al. 2018; Alonso-Navarro et al. 2025; Downar et al. 2025). Despite growing recognition of stress as a modulating factor in ET, its role in symptom variability remains poorly understood, with no studies to date directly addressing this relationship.

This explorative study aimed to examine the relationship of psychopathological and stress-related symptoms with clinical features in ET patients. Gaining insight into the complex interplay between neuropsychiatric symptoms, stress-related factors, and tremor variability is essential for developing personalized therapeutic strategies and elucidating the mechanisms underlying ET heterogeneity (Holding and Lew 2015; Berry et al. 2024; Varghese et al. 2024; Kapinos and Louis 2024).

## Materials and methods

### Participants

We consecutively enrolled 47 subjects diagnosed with ET according to the current consensus criteria (Bhatia et al. 2018) (Table 1). Participants were recruited from the outpatient clinic for movement disorder of the Policlinico Umberto I, Department of Human Neurosciences, Sapienza University of Rome, Italy. ET was defined as an isolated bilateral upper limb action tremor lasting at least three years, with or without tremor in other regions, and with or without additional neurological signs of uncertain significance, also known as ‘soft signs’ (Bhatia et al. 2018; Paparella et al. 2021, 2024a, b; Angelini et al. 2022). Exclusion criteria included neurological signs indicative of alternative diagnoses, such as dystonia, ataxia, or parkinsonism (Postuma et al. 2015; Bhatia et al. 2018; Angelini et al. 2022; Paparella et al. 2024b; Albanese et al. 2025). Patients undergoing tremor treatment were evaluated after drug withdrawal, which was accomplished by tapering doses during the week preceding assessment. Medications were discontinued 24 hours before evaluation for propranolol and benzodiazepines, and 48 hours prior for primidone and topiramate (Paparella et al. 2018, 2020, 2024b; De Biase et al. 2022; Angelini et al. 2022). Patients presenting clinically relevant psychiatric symptoms during the interview were subsequently referred to the department’s psychiatric service. All participants provided informed consent before participating. The study was approved by the local institutional review board, conducted in accordance with international safety guidelines and adhered to the ethical standards of the Declaration of Helsinki.

**Table 1:** Demographic and clinical data in patients with essential tremor (ET)

Demographic and clinical data	ET sample
Sex (F: M)	24:23
Age [mean ± SD]	65.47 ± 12.64
Age at onset [mean ± SD]	47.96 ± 19.74
Familiarity [N (%)]	26 (55.32%)
Years of education [mean ± SD]	9.67 ± 5.11
Caffeine users [N (%)]	35 (74.5%)
Smokers [N (%)]	26 (27.7%)
Tremor treatment	
Propranolol [N (%)]	18 (38.3%)
BDZ [N (%)]	10 (21.3%)
Primidone [N (%)]	0 (0.0%)
Topiramate [N (%)]	2 (4.2%)
Others [N (%)]	10 (21.3%)
Onset site	
Head [N (%)]	7 (14.89%)
Unilateral upper limb [N (%)]	16 (34.04%)
Upper limbs symmetrical [N (%)]	10 (21.28%)
Upper limbs asymmetrical [N (%)]	14 (29.79%)
Tremor distribution	
Head tremor [N (%)]	23 (48.93%)
Face tremor [N (%)]	11 (23.4%)
Voice tremor [N (%)]	20 (42.55%)
Upper limbs tremor [N (%)]	47 (100%)
Lower limbs tremor [N (%)]	13 (27.66%)
Rest tremor [N (%)]	29 (61.7%)
TETRAS [mean ± SD]	
TETRAS_ADL_	13.72 ± 9.15
TETRAS_p_	22.37 ± 10.58
TETRAS_TOT_	36.10 ± 18.47
SARA [mean ± SD]	2.04 ± 2.12
MDS-UPDRS items 3.4–3.8 [mean ± SD]	0.95 ± 1.82
MOCA TOT [mean ± SD]	25.87 ± 2.35
QoL [mean ± SD] HR-QoL [mean ± SD]	76.28 ± 16.43 67.76 ± 24.04

F: female; M; male. Age and age at onset are expressed in years. TETRAS: The Essential Tremor Rating Assessment Scale, including the performance (TETRAS_P_) and the activity of daily living subscales (TETRAS_ADL_). SARA: scale for the assessment and rating of ataxia. MoCA: Montreal Cognitive Assessment. QoL: quality of life, HR-QoL: health related quality of life

### Clinical evaluation

We collected key demographic and clinical data from participants (Table 1), including family history of tremor or other neurological disorders, age at tremor onset, disease duration and habits related to caffeine, tobacco, and alcohol consumption, as well as tremor response to alcohol. All patients underwent a comprehensive neurological examination. Tremor severity was assessed using the Essential Tremor Rating Assessment Scale (TETRAS) (Elble et al. 2012), which includes the performance subscale (TETRAS_P_) and the activities of daily living subscale (TETRAS_ADL_). Action and rest upper limb tremor scores were calculated from individual TETRAS_P_ items as described elsewhere [6]. To evaluate subtle cerebellar or parkinsonian signs, we used the Scale for the Assessment and Rating of Ataxia (SARA) (Schmitz-Hübsch et al. 2006), and the Movement Disorder Society Unified Parkinson’s Disease Rating Scale (MDS-UPDRS) Part III (Goetz et al. 2008). Impaired tandem gait was identified when patients made more than one misstep during the task but still completed it without a clearly pathological gait (Angelini et al. 2022). Patients scoring at least 1 on MDS-UPDRS items 3.4–3.8 were classified as having subtle bradykinesia (Angelini et al. 2022; Paparella et al. 2023, 2024a; Bologna et al. 2023). The presence of questionable dystonic postures was also clinically evaluated (Bhatia et al. 2018; Paparella et al. 2024a; Albanese et al. 2025). Cognitive function was briefly screened using the Montreal Cognitive Assessment (MoCA) (Nasreddine et al. 2005). Finally, patients self-reported their overall quality of life (QoL) and health-related quality of life (HR-QoL).

### Psychiatric assessment

A diagnostic psychiatric assessment was conducted by a psychiatrist through a clinical interview, focused on the main criteria of the Diagnostic and Statistical Manual of Mental Disorders 5th edition (DSM-5). We also adopted the Hospital Anxiety and Depression Scale (HADS) to additionally score anxiety and depressive symptoms, with scores ≥ 8 on either or both anxiety and depressive subscales being considered suggestive of clinically relevant symptoms (Stern 2014).

### Evaluation of stress, stress-related symptoms and resilience

We used the Perceived Stress Scale (PSS) (Cohen et al. 1983), using the following cutoff (based on previous research using PSS): scores of 0–13 as suggestive of low perceived stress, scores ≥ 14 as suggestive of moderate or high perceived stress (Anandhalakshmi et al. 2016; Philpott et al. 2022; Rajanayagam et al. 2023). Participants were also assessed using the Insomnia Severity Index (ISI) (Castronovo et al. 2016), with scores of 0–7 suggesting no clinically significant insomnia, of 8–14 suggesting subthreshold insomnia, of 15–21 suggesting moderate insomnia, and of 22–28 suggesting severe insomnia (Castronovo et al. 2016). We also investigated through the clinical interview the presence of vivid dreams or involuntary movements during sleep that might suggest restless leg syndrome or REM behavior disorders. Resilience was assessed using the Brief Resilience Scale (BRS), a 6-item tool focused on the ability to bounce back or recover from stress (Smith et al. 2008; Laudadio et al. 2011); scores lower than 3 have been considered as suggestive of low resilience, while scores above 4.3 have been considered as suggestive of high resilience, based on previous evidence (Smith et al. 2013). Finally, the Alcohol Use Disorders Identification Test - Consumption (AUDIT-C) scale was used for the identification of alcohol abuse disorders, with scores ≥ 5 in males and ≥ 4 in females indicative of risky alcohol consumption (Scafato et al. 2010; van Gils et al. 2021).

### Statistical analysis

Data distribution was evaluated visually and with the Shapiro-Wilk test. Due to the non-normal distribution of many variables, non-parametric tests were applied. Participants were divided into subgroups based on the presence of psychiatric disorders identified during the clinical interview and/or by HADS scores ≥ 8 on either or both subscales, and based on standardized cut-off values from the PSS, ISI and BRS scales. Categorical variables—including sex, presence of tremor in the head, face, voice, lower limbs, rest tremor, and soft signs such as questionable bradykinesia, dystonia, and impaired tandem gait—were presented as frequencies and compared between subgroups using Fisher’s exact test. The Mann-Whitney U test assessed between-groups differences in relation to quantitative clinical data, such as tremor scores, severity of action and rest upper limb tremor, SARA, MoCA, and quality of life scores. Spearman’s correlation analyses on the whole sample evaluated relationships among demographic (e.g. age, age at tremor onset), tremor (TETRAS_TOT_, TETRAS_P_, TETRAS_ADL_, upper limbs action and rest tremor severity, SARA, UPDRS items 3.4–3.8 and MoCA scores) and psychiatric and stress-related variables (HADS, PSS, ISI, BRS scores). Results are reported as mean ± standard deviation. Statistical significance was set at *P* < 0.05, with multiple comparisons corrected using the false discovery rate (FDR) (Benjamini and Hochberg 1995). Finally, for the self-reported measures, we tested the internal consistency using the Cronbach’s alpha, with values > 0.7 indicating good internal consistency. Data analysis was carried out using STATISTICA^®^ (TIBCO Software Inc., Palo Alto, California, USA).

## Results

Table 1 summarizes the main clinical and demographic data of the 47 patients. The sample included 24 females (51.1%), with a mean age of 65.47 ± 12.64 years, a mean age at tremor onset of 47.96 ± 19.74 years, and a mean disease duration of 17.51 ± 15.65 years. Twenty-six patients (55.3%) reported a family history of tremor.

### Clinical neurological evaluation

All patients had bilateral upper limb action tremor; additionally, 23 (48.1%) had head tremor, 11 (23.4%) face tremor, 20 (42.6%) voice tremor, and 13 (27.7%) lower limb tremor. Rest tremor was observed in 29 patients (61.7%), and questionable bradykinesia in 11 (23.4%), though none met full criteria for parkinsonism (Postuma et al. 2015). Three patients (6.4%) showed questionable dystonic posturing, and 16 (34%) exhibited mild impaired tandem gait. Cognitive function, assessed by MoCA, averaged 25.87, with 18 patients (38.3%) presenting scores lower than 26, while quality of life scores were moderate (QoL: 76.28 ± 16.43; HR-QoL: 67.76 ± 24.04). Further clinical scale results are detailed in Table 1.

### Psychiatric assessment

We observed that 23 out of 47 patients (48.9%) had ongoing psychiatric disorders emerging from the psychiatric interview (Supplementary Table 1). The most common psychiatric conditions among the patients included: depressive disorders (14 patients, 29.8%), anxiety disorders (11 patients, 23.4%), and adjustment disorders with anxiety and/or depressed mood (5 patients, 10.6%). Two patients (4.2%) also had a personality disorder (notably Cluster B types), while one patient (2.1%) had a bipolar I disorder. Moreover, 10 patients (24.4%) had a documented history of past psychiatric diagnoses, including depressive and anxiety disorders (4 patients, 8.6%), adjustment disorder with anxiety and depressed mood (2 patients, 4.3%), eating disorders (2 patients, 4.3%), alcohol abuse disorder (1 patient, 2.1%), bipolar I disorder (1 patient, 2.1%), and cluster B personality disorder (1 patient, 2.1%). Finally, a family history of psychiatric conditions was noted in 6 patients (12.7%). The mean HADS scores (global scale Cronbach’s alpha 0.76) were 6.08 ± 3.01 for anxiety and 5.14 ± 3.53 for depression (Table 2). Twenty patients (42.5%) had abnormal values (≥ 8) for anxiety and/or depression. Although the subgroup of patients with psychiatric disorders had a slightly younger age (61.58 ± 13.08 vs. 70.28 ± 10.46 years), a slightly less severe tremor (TETRAS_TOT_: 29.96 ± 15.43 vs. 43.69 ± 19.43; TETRAS_P_: 18.96 ± 8.65 vs. 26.59 ± 11.39; TETRAS_ADL_: 11 ± 8.2 vs. 17.09 ± 9.25; rest tremor severity 1.04 ± 1.39 vs. 2.12 ± 2.07), and a lower prevalence of questionable bradykinesia (3 out of 26 vs. 8 out of 21) compared to those without any psychiatric disorders, these differences did not survive after FDR correction (all *P*_adj > 0.05).

**Table 2:** Psychiatric scores and stress-related disturbances scores in patients with essential tremor (ET)

Psychiatric and stress-related disturbances scores	ET
HADS [mean ± SD]	
Total anxiety	6.08 ± 3.01
Total depression	5.14 ± 3.53
PSS [mean ± SD]	14.53 ± 6.71
ISI [mean ± SD]	7.15 ± 5.2
BRS [mean ± SD]	3.55 ± 0.77
AUDIT-C [mean ± SD]	1.53 ± 1.44

HADS: Hospital Anxiety and Depression Scale. PSS: Perceived Stress Scale. ISI: Insomnia Severity Index. BRS: Brief Resilience Scale. AUDIT-C: Alcohol Use Disorders Identification Test - Consumption scale. Data are indicated as mean ± standard deviation

### Evaluation of stress, stress-related symptoms and resilience

Mean PSS score was 14.53 ± 6.71 (Cronbach’s alpha 0.79, Table 2), with 31 patients (65.9%) showing moderate/high levels of perceived stress and 16 patients (34%) showing low levels of perceived stress based on the described cutoff scores (Anandhalakshmi et al. 2016; Rajanayagam et al. 2023). We found that face and lower limbs tremor were less frequently observed among patients showing moderate/high levels of perceived stress (face tremor: 3 out of 31 and 8 out of 16, *P* = 0.003; lower limbs: 4 out of 31 and 9 out of 16, *P* = 0.002). No other significant differences were observed with respect to demographic variables, including age, or neurological symptoms (all *P* values > 0.05).

**Fig. 1:**
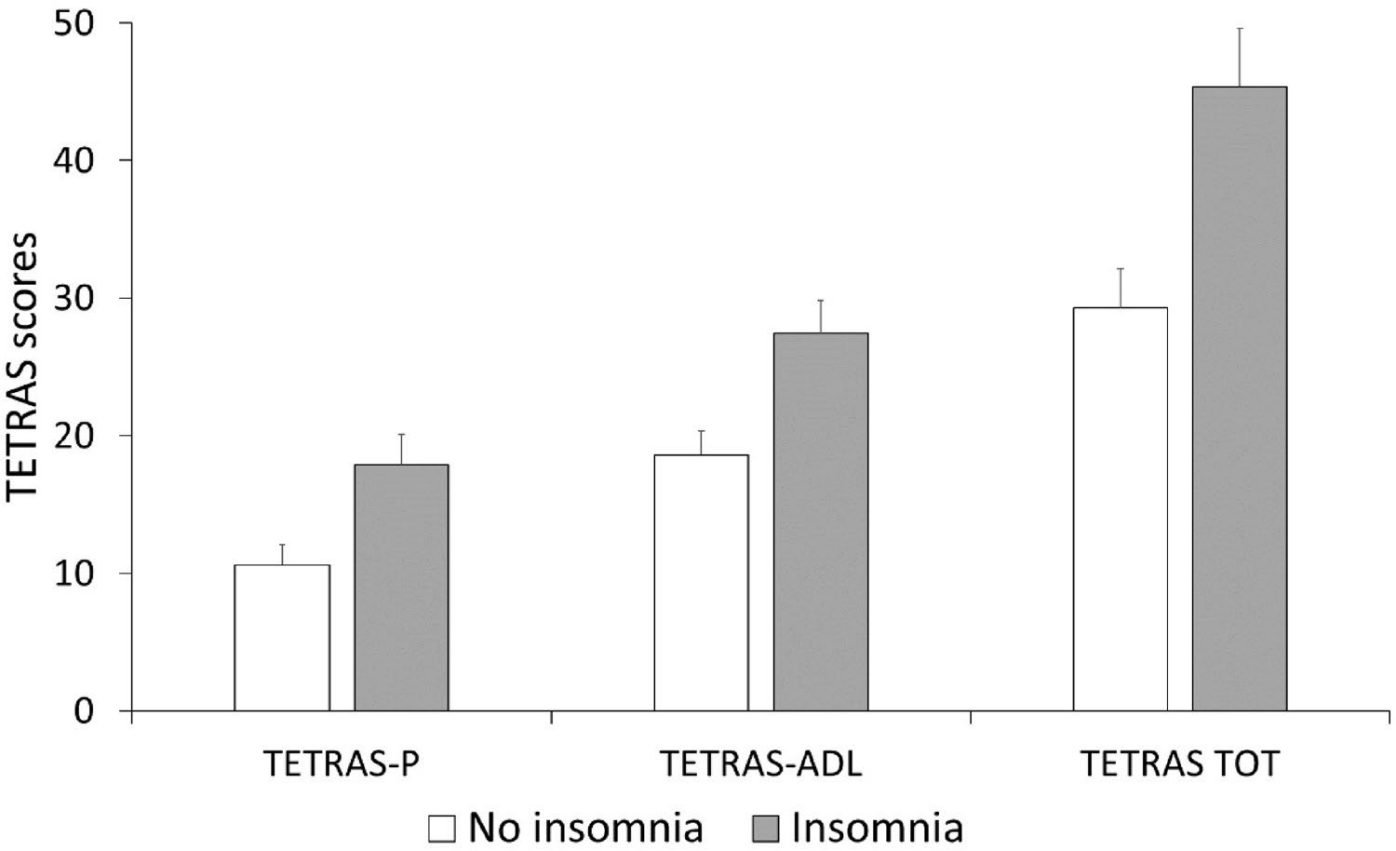
Essential Tremor Rating Assessment Scale (TETRAS) in essential tremor (ET) patients with and without insomnia, as evaluated by the Insomnia Severity Index (ISI). TETRAS-P: performance subscale. TETRAS-ADL: activity of daily living subscale. TETRAS-TOT: total scores. Bars indicate mean values, error bars standard error of the mean

**Fig. 2:**
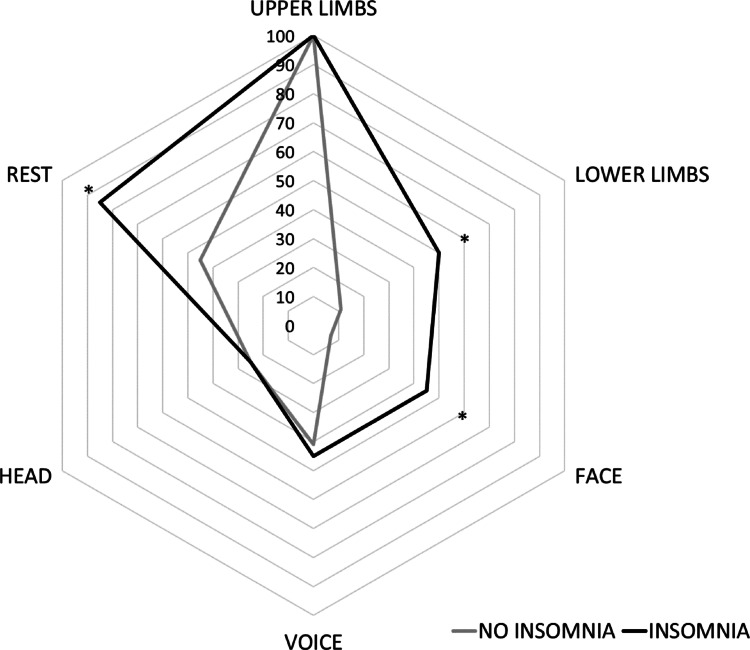
Percentage of essential tremor (ET) patients with and without insomnia (based on an Insomnia Severity Index [ISI] score < 8 or ≥ 8), showing tremor in the upper limbs, lower limbs, head, face, voice, and at rest. Tremor in the upper and lower limbs refers specifically to action tremor. Statistically significant differences, as determined by Fisher’s exact test, are indicated by asterisks

The mean ISI score was 7.15 ± 5.2 (Cronbach’s alpha 0.85, Table 2). Namely, 20 patients (42.55%) reported symptoms of insomnia based on the described cutoff scores, 17 had clinical subthreshold insomnia, and 3 presented with moderate insomnia, while no cases of severe insomnia were observed. A similar proportion of patients between those with and without insomnia (5 out of 20 and 6 out of 27) reported the presence of vivid dreams, but none of them had a clinical diagnosis of REM behavior disorders. When comparing patients with (ISI ≥ 8) and without insomnia (ISI < 8), we found higher TETRAS_TOT_ (*P* = 0.005), TETRAS_P_ (*P* = 0.008) and TETRAS_ADL_ scores (*P* = 0.007) in the former subgroup (Fig. 1). We found a more severe rest upper limb tremor (2.4 ± 2.04 vs. 0.87 ± 1.26, *P* = 0.004), and face, lower limbs and rest tremor were more prevalent in patients with insomnia compared to those without. Specifically, among patients without insomnia, 7.4% exhibited facial tremor, 11.1% had lower limb tremor, and 44.4% experienced rest tremor, while among those with insomnia the prevalence of these tremor types was substantially higher: 40% for facial tremor (*P* = 0.003), 45% for lower limb tremor (*P* = 0.004), and 80% for rest tremor (*P* = 0.005) (Fig. 2). Finally, MoCA scores were slightly lower in patients with insomnia (24.95 ± 2.56 vs. 26.55 ± 1.96), although this difference did not remain significant after FDR correction. No other significant differences, including age, age at onset and disease duration (all *P* values > 0.05), were observed in relation to neurological symptoms.

ET patients demonstrated a mean BRS score of 3.55 ± 0.77 (Cronbach’s alpha 0.75) [66] (Table 2). Specifically, 17 patients showed low resilience, 20 normal resilience, 10 high levels of resilience. When comparing participants with low vs. normal/high resilience, we did not find any differences in terms of age or other demographic data (all *P* values > 0.05). A positive familiar history for tremor was more frequent among patients with normal/high resilience (5 out of 17 and 21 out of 30, *P* = 0.003). Conversely, questionable bradykinesia was more frequent in patients with low resilience (8 out of 17 vs. 3 out of 30, *P* = 0.006). Table 2 also depicted the AUDIT-C scores (Cronbach’s alpha 0.01). Notably, only one patient had an AUDIT-C score suggestive of hazardous alcohol consumption (van Gils et al. 2021).

### Correlation analysis

**Fig. 3:**
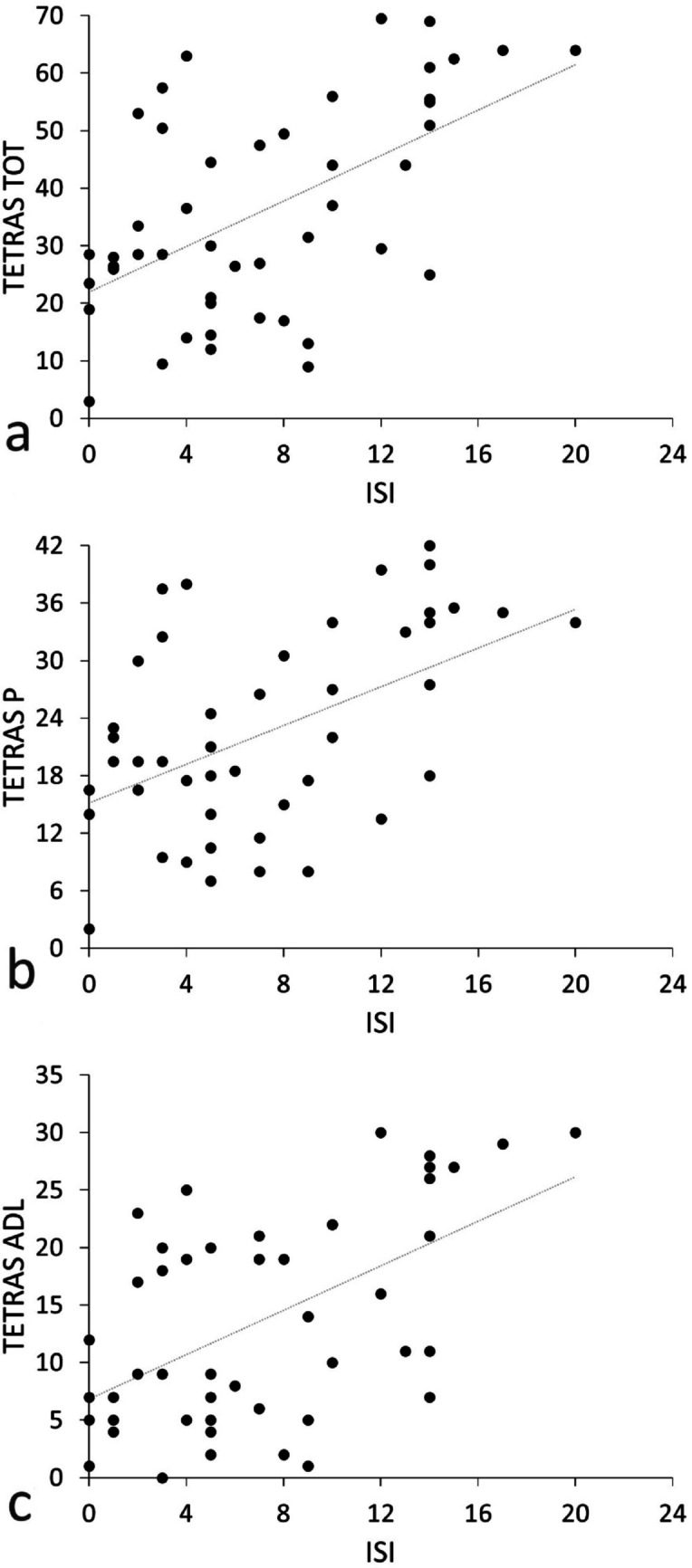
Correlation between the Insomnia Severity Index (ISI, X axes) and The Essential Tremor Rating Assessment Scale total (TETRAS TOT, Y axis, Panel A) performance (TETRAS P, Y axis, Panel B) and ADL subscores (TETRAS ADL, Y axis, Panel C)

Correlation analysis demonstrated a positive correlation between the TETRAS_TOT_ scores and ISI scores (*R* = 0.48, *P* < 0.001) (Fig. 3). Again, ISI scores correlated with both TETRAS_P_ and TETRAS_ADL_ scores (*R* = 0.42, *P* = 0.002, *R* = 0.5, *P* < 0.001) (Fig. 3). These results overall indicate that greater levels of insomnia are associated with higher tremor severity, both in terms of motor symptoms and the impact of tremor on daily functioning. We found no other significant correlations between clinical data.

## Discussion

In this study, we systematically examined the relationship of psychopathological and stress–related symptoms with clinical variability in ET patients. Using specialist interviews and standardized scales, we found in a portion of patients the presence of **concurrent** psychiatric disorders, including mood and personality disorders. Some patients also exhibited elevated perceived stress levels, with those experiencing higher stress less likely to have tremor affecting body regions beyond the upper limbs. A certain amount of the observed patients reported symptoms of insomnia, and those affected exhibited higher tremor scores compared to patients without insomnia. Further, a positive association was observed between tremor severity and insomnia. These findings provide insights into the phenotypic clinical variability of ET and may enhance clinical assessment and understanding of its pathophysiology.

In line with previous findings, we confirmed the presence of psychiatric comorbidities in ET (Fabbrini et al. 2012; Louis et al. 2016; Louis 2016; Monin et al. 2017; Huey et al. 2018; Bologna et al. 2019; Ratajska et al. 2022; Angelini et al. 2022), including depressive, anxiety and adjustment disorders, as well as bipolar and personality disorders, with more than 40% of patients with abnormal values for anxiety and/or depression as assessed by standardized scales. Clinical studies on this topic, although showing some variability (Aslam et al. 2017), have reported depression, apathy, anxiety, and personality disturbances in 8.5% up to 54% of ET patients (Monin et al. 2017; Bologna et al. 2019; Angelini et al. 2022). As in earlier reports (Bologna et al. 2019; Angelini et al. 2022), in the present study a clinical interview conducted by a trained psychiatrist was performed, in addition to the use of validated self-report scales. We did not observe a significant association between the presence of psychiatric disorders and tremor severity. It is possible that psychiatric disorders in ET not only represent possible distinct phenomena or possible psychological reactions to the overall disease burden, but rather they may constitute a manifestation of ET itself (Lenka et al. 2017; Bologna et al. 2019). In this context, the cerebellum likely plays a central role. Indeed, growing neuroanatomic and neuroimaging evidence demonstrated that the cerebellum, especially the vermis (Stoodley and Schmahmann 2010), is reciprocally connected with the limbic system, it is known to alter affective processes and it is involved in neuropsychiatric disorders (Fitzgerald et al. 2008; Hoppenbrouwers et al. 2008; Stoodley and Schmahmann 2010; Xu et al. 2018; Hilber et al. 2019; Paparella et al. 2024b).

An innovative aspect of the present study lies in the investigation of perceived stress in ET patients and its relationship with tremor variability and related features. Although the relationship between stress and tremor is widely acknowledged, this is mainly based on empirical evidence supporting stress as an influencing factor in tremor. However, no previous studies have specifically addressed this topic using standardized stress measures. One prior report investigated the role of stress in veterans affected by ET, concluding that chronic stress contributed not only to psychological comorbidities such as anxiety and depression, but also, in some cases, directly or indirectly induced tremor (Handforth and Parker 2018). Additional data comes from recent studies assessing tremor changes following SARS-CoV-2 infection and related psychological stress (Passaretti et al. 2022; Costa et al. 2023, 2024; Pakan et al. 2024). Moreover, some pharmacological reports suggested that the efficacy of tremor medications may be partly attributed to their effects on stress and stress-related disorders in ET (Gengo et al. 1986, p. 19; Paparella et al. 2018; Alonso-Navarro et al. 2025; Downar et al. 2025). To our knowledge, this is the first study to employ the PSS, a widely used instrument for measuring perceived stress, in patients with ET. The majority of our patients showed moderate/high levels of perceived stress (based on the described PSS cut-off scores). Subgroup analysis, based on binary categorization of patients, failed to show significant differences in overall tremor severity between patients with high vs. low perceived stress. However, we observed that patients with higher perceived stress levels were less likely to exhibit tremor in the face and lower limbs compared to those with lower perceived stress. This observation expands upon one previous research that, as opposed to us, indicated that patients with more severe or those with vocal tremor experience higher levels of stigma and psychological distress (O’Suilleabhain et al. 2023). As theoretical considerations, our findings raise the possibility that individuals with tremor affecting visible or functionally critical areas (such as the face and legs) may have developed compensatory psychological or behavioural strategies over time, which could enable them to better manage stress and to cope with the psychological burden associated with the visibility and functional impact of their symptoms, ultimately resulting in lower reported levels of perceived stress.

In parallel, our data also shed light on the role of resilience, conceptualized as the ability to adapt successfully in the face of adversity, trauma, or stress. Within the sample, 17 patients out of 47 showed low resilience, and patients who exhibited higher resilience were more likely to report a positive family history of tremor. This finding may reflect another form of adaptive coping—possibly developed early in life through long-term exposure to the condition within the family—leading to greater acceptance, understanding, and psychological preparedness to manage the challenges associated with ET. Finally, the observation that specific soft signs, including questionable bradykinesia, were more frequent in ET patients with low resilience needs to be addressed in future study on larger samples.

Concerning stress-related symptoms, another important finding in our study is that over 40% of patients reported symptoms of insomnia, as evaluated by the ISI. Notably, patients with insomnia had comparable age, age at onset, and disease duration to those without insomnia, however they exhibited higher tremor scores, including total (TETRAS_TOT_), performance (TETRAS_P_), and activities of daily living (TETRAS_ADL_) scores, compared to those without symptoms of insomnia. Furthermore, tremor involving the face, lower limbs, and at rest was more frequently observed in patients with insomnia. These individuals also showed slightly lower cognitive scores than those without sleep disturbances. Consistently, we identified a significant correlation between insomnia severity and tremor severity: the more severe the insomnia, the higher the TETRAS scores.

While sleep disturbances in ET are well-documented, few studies have examined their association with variability in clinical presentation (Barut et al. 2015; Jiménez-Jiménez et al. 2021; Sringean 2024). Our findings suggest a possible bidirectional relationship between motor symptoms and sleep disturbances, where more severe or disabling tremor may contribute to poorer sleep quality, or conversely, insomnia may exacerbate the functional and subjective experience of tremor. The findings may also be interpreted through a pathophysiological lens, suggesting a potentially shared mechanism between tremor and insomnia. Prior research has highlighted the role of the locus coeruleus (LC) in sleep regulation, with LC dysfunction linked to insomnia (Li et al. 2022; Mortazavi et al. 2025). Additionally, degeneration of the LC has been reported in ET patients, possibly associated with Lewy body accumulation and cerebellar degeneration—both of which could impact motor control and sleep regulation (Ghanem et al. 2024; Fang et al. 2025). Our results contrast with a previous study that did not find a relationship between LC integrity and sleep quality (Liu et al. 2024). However, methodological differences, including the use of different clinical scales and the inclusion of both ET and Parkinson’s disease (PD) patients, may account for the discrepancy. Finally, a similar proportion of patients with and without insomnia symptoms in our sample reported experiencing vivid dreams, and none had a clinical diagnosis of REM sleep behaviour disorder. This suggests that insomnia was likely not due to premotor symptoms indicative of a potential conversion to PD. However, the present topic requires further investigation.

The observed association between sleep disturbances and tremor severity may have clinical relevance. First, the findings underscore the importance of systematically assessing and addressing sleep quality in patients with tremor disorders. Again, it could help explain why sodium oxybate—used to treat narcolepsy—has been reported to reduce tremor severity in ET patients (O’Flynn et al. 2023). The results may also indicate that interventions aimed at improving sleep, including non-pharmacological approaches, could indirectly alleviate tremor in patients with ET, similarly to what has been observed in PD, where mindfulness-based approaches have been shown to reduce psychological distress and improve clinical symptoms (van der Heide et al. 2021). The slightly lower MoCA scores observed in patients with insomnia are consistent with recent longitudinal evidence suggesting that sleep disturbances may predict cognitive decline in ET (Tsapanou et al. 2024), and further underscore the importance of further research into the interplay between sleep, cognition, and motor symptoms in this condition. Finally, the high rates of neuropsychiatric symptoms and stress-related disorders observed in patients with ET underscore the importance of thoroughly evaluating non-motor symptoms when considering both candidacy and postoperative outcomes of neurofunctional tremor treatments such as deep brain stimulation (DBS) (Deuschl et al. 2006; Lang et al. 2006; Ferreira Felloni Borges et al. 2023; Zhang et al. 2024; Berry et al. 2025).

There are some confounding factors and limitations to be taken into consideration. Although the diagnosis of ET was primarily clinical and not all patients underwent DaTSCAN imaging, all participants have been followed at our specialized neurological outpatient clinic for several years. Ongoing follow-up strengthens diagnostic accuracy and reduces misclassification risk. Furthermore, considering that tremor is an highly variable symptom, assessments were consistently performed at the same time of day and under similar conditions for all patients. Additionally, while tremor treatments were discontinued before assessment, the potential impact of psychiatric medications should be considered when interpreting the results. However, three patients were taking antidepressants and two were on gabapentinoids, which were not discontinued prior to the assessment. While the frequency of soft plus signs such as rest tremor, impaired tandem gait, or questionable bradykinesia in our sample aligns with clinical experience (Bhatia et al. 2018; Pandey and Bhattad 2021; Erro et al. 2022, 2024; Lalli and Albanese 2024), the frequency of questionable dystonic posturing appears relatively low. However, this is consistent with recent observations demonstrating that questionable dystonic postures in ET are difficult to identify and are characterized by high inter-rater variability (Paparella et al. 2024a). Although we used some self-reported scales for psychiatric assessment, they overall demonstrated good internal consistency, as indicated by their Cronbach’s alpha values. Similarly, the rationale for assessing sleep via self-report was primarily based on feasibility and resource considerations, as objective measures such as polysomnography were not available for all participants. Additional limitations include the absence of objective markers of stress, such as cortisol sampling, and the lack of data on cognitive reserve in patients. Other constraints of the study are the relatively small sample size and the absence of a healthy control group or a disease control group. This prevents us from determining whether the observed associations, particularly between insomnia and tremor severity, are specific to ET or may also occur in other tremor syndromes or neurodegenerative conditions. Nonetheless, as an exploratory study, it offers preliminary insights that warrant validation in larger, longitudinal cohorts with appropriate controls, that will be crucial to confirm the specificity of our findings and to better elucidate the underlying pathophysiological mechanisms. These future studies will be also address why we observed an higher prevalence of questionable bradykinesia in patients with no anxiety nor depression.

In conclusion, we here revealed psychiatric disorders, elevated perceived stress and some stress-related symptoms, including insomnia, among patients with ET. Although no direct link was found between stress or psychiatric comorbidities and tremor severity, the association between tremor and insomnia suggests a possible shared pathophysiological mechanism between the two conditions. The present findings contribute to reinforce the understanding of ET as a multisystem disorder involving both motor and non-motor domains (Louis 2011; Bologna et al. 2019; Paparella et al. 2021; Colella et al. 2023; Birreci et al. 2025). Further research is needed to validate these results and explore the longitudinal interaction between neurological and psychiatric features in ET.

## Data Availability

The data supporting this study’s findings are available on request from the corresponding author.

## Abbreviations

AUDIT-C: Alcohol use disorders identification test – consumption
ADL: Activity of daily living
BRS: Brief resilience scale
HADS: Hospital anxiety and depression scale
HR-QoL: Health related quality of life
ISI: Insomnia severity index
LC: Locus coeruleus
MoCA: Montreal cognitive assessment
MDS-UPDRS: Movement Disorder Society-sponsored revision of the Unified Parkinson’s Disease Rating Scale
PD: Parkinson’s disease
PSS: Perceived stress scale
QoL: Quality of life
SD: Standard deviation
TETRAS: The essential tremor rating assessment scale
SARA: Scale for the assessment and rating of ataxia
